# Nutraceutical Properties of *Medicago sativa* L., *Agave* spp., *Zea mays* L. and *Avena sativa* L.: A Review of Metabolites and Mechanisms

**DOI:** 10.3390/metabo12090806

**Published:** 2022-08-28

**Authors:** Tannia A. Quiñones-Muñoz, Socorro J. Villanueva-Rodríguez, Juan G. Torruco-Uco

**Affiliations:** 1 Consejo Nacional de Ciencia y Tecnología (CONACYT)—Centro de Investigación y Asistencia en Tecnología y Diseño del Estado de Jalisco (CIATEJ), Av. Normalistas 800, Colinas de la Normal, Guadalajara C.P. 44270, Mexico; 2 Centro de Investigación y Asistencia en Tecnología y Diseño del Estado de Jalisco (CIATEJ), Av. Normalistas 800, Colinas de la Normal, Guadalajara C.P. 44270, Mexico; 3 Tecnológico Nacional de Mexico/Instituto Tecnológico de Tuxtepec, Calzada Dr. Víctor Bravo Ahuja, 561, Col. Predio el Paraíso, San Juan Bautista Tuxtepec C.P. 68350, Mexico

**Keywords:** crops, bioactivities, functional food, antioxidant compound, bioactive extracts

## Abstract

Plants are the main sources of bioactive compounds (nutraceuticals) that function under different mechanisms of action for the benefit of human health. Mexico ranks fifth in the world in biodiversity, offering opportunities for healthy food. An important variety of crops are produced in the state of Hidalgo, e.g., based on the 2021 production, alfalfa, oats, maguey, and corn. The present review presents the latest findings of these crops, regarding the benefits they provide to health (bioactivity, nutraceuticals), and presents the compounds and mechanisms identified by which the benefit is provided. The knowledge compiled here is for the benefit of the recovery of the crops, the recognition of their bioactivities, in search of identifying the best routes of action for prevention, treatment and possible cure of chronic degenerative diseases (thereby promoting crop valorization). Exhaustive bibliographic research was carried out by means of engines and scientific databases. Articles published between 2001 and 2022 that included specific keywords (Scopus, EMBASE, EBSCO, PubMed, Science Direct, Web of Science, Google Scholar). Outstanding activities have been identified for the compounds in the crops, such as antiinflammatory, anticholesterolemic, antihypertensive, antidiabetic, anticancer, antimicrobial, antioxidant, and chelating. The compounds that provide these properties are total phenols, phenolic acids, tannins, anthocyanins, carotenoids, iso-flavones, phytosterols, saponins, fructans, glycosides, glucans, avenanthramides, and polysaccharides.

## 1. Main Crops of Hidalgo, Mexico, and Their Use in the Gastronomy and Health

Bioactive compounds present in plants (more than 2 million identified), also called phytochemicals, are produced by the defense system that is activated by the presence of biotic and abiotic stress conditions; in addition to improving the general health status of plants, they participate in molecular signaling and in plant-environment interaction [1,2]. The biosynthesis of these secondary metabolites in plants has specific localization organs from which they are transported to the whole plant and the main storage parts (vacuoles) [3]. The recovery of these compounds can be developed from any part of the plant (roots, stems, leaves, somatic embryos, callus, flowers) but it should be considered that they can vary in type and concentration depending on the part used, and therefore, in the bioactivity detected. Bioactivities can be developed from various mechanisms of action, both beneficial to the plant and to humans. Due to the great use that can be made of these compounds, it is necessary to know in depth the factors that influence their production, recovery and maintenance techniques, their mechanisms of action, in addition to an emerging factor in recent years, climate change, which without much explanation, could modify the advances already known about these compounds, their natural sources and possible changes of adaptation.

Mexico ranks fifth in the world in biodiversity, offering opportunities for healthy food. Ninety percent of the population of the state of Hidalgo, Mexico, works in the agricultural sector and, what makes the sector a strategic area for said state, as well as for food sovereignty of Mexico. Hidalgo contributes 2.9% of the national volume of agricultural products [4]. Grain corn has a production value for the year 2021 of $2,756,711.47, green forage oats a value of $104,610.10, of a total annual value for the state of $5,081,511.37 (thousands of Mexican pesos); therefore, the importance of these crops (agricultural year) in production and economy is strengthened. The production values of alfalfa and maguey pulquero, the most important production crops (perennial), are greatest, amounting to $1,448,552.37 and $576,812.51, respectively, of a total annual amount of $2,601,538.05 (thousands of Mexican pesos). The third and fourth place in terms of production value (perennial) have an economic value of $74,165.64 and $187,940.07 (thousands of Mexican pesos), which is a lower production value than alfalfa and pulquero maguey (reported as produced honey water) [5]. The criteria considered for the selection of the crops under review at the bioactive level were focused on the annual production and the economic value they represent, as shown in Table 1 and in the description of the previous lines.

On the other hand, Hidalgo stands out for the diversity of its gastronomy characterized by the presence of dishes made with exotic ingredients, traditionally prepared with sophisticated culinary techniques. Within the list of representative foods, the predominant crops in the region are widely present, maguey and corn. Worms are obtained from the maguey; mixiotes (enchilada meat) and ximbo (cooked meat rolled in stalk) are cooked with a film obtained from the stalk; gualumbos or quiotes are obtained from their flowers; pastes, zacahuil (corn), peanut tamale, guajolotes (telera cakes with beans and enchiladas), moles, tecoquitos, bocoles, and molotes are made from corn. Another regional dish is escamoles, which are the larvae or roe of the scale ant [6].

As can be seen in the preceding paragraphs, in the State of Hidalgo, Mexico, important crops are identified by production and tradition, which have an important impact on the economic development of the State. The present review presents the latest findings of these crops, regarding the benefits they provide to health (bioactivity, nutraceuticals), and presents the compounds and mechanisms identified by which the benefit is provided. No other similar work can be found, neither for the same crops nor with the detailed analysis of properties, mechanisms, concentrations, and types of samples (extracts, plant portions), as discussed in the present work. The knowledge compiled here is, in addition, for the benefit of the recovery of the crops, the recognition of their bioactivities, in search of identifying the best routes of action for prevention, treatment, and possible cure of chronic degenerative diseases (thereby promoting their valorization). In this context, the objective of the present work is to identify the recent findings regarding the main crops of the State of Hidalgo, Mexico, their bioactive properties, and the compounds (nutraceuticals) that develop them, for their better use in the prevention, treatment, or cure of chronic degenerative diseases.

## 2. Nutrients and Bioactive Compounds in Important Crops

The main crops sown in Hidalgo have a variety of applications in the diet, mainly nutrients and bioactive compounds. Considering and valuing these properties that crops contribute to human health would strengthen the appropriation of regional cultivation, influencing food sovereignty and regional development. By taking advantage of all these crops in an integral way, production would be boosted, generating direct and indirect economic, social, nutritional, and cultural benefits, among others.

It is important to remember that the biological activities determined are not exclusive to a molecule per se (unless the solution has been so prepared) considering its physicochemical and structural characteristics (conjugated double bonds, number and position of methyl and hydroxyl groups), since in extracts, it can be attributed to effects of complexes formed with the individual molecules (synergistic, additive or antagonistic) and to the sensitivity of each molecule or complex to different analytical techniques (free radical inhibition, metal reduction). The bioactive properties discussed below may be a response of individual properties or interactions, depending on the sample analyzed.

### 2.1. Alfalfa (Medicago sativa *L.*)

According to the Statistical Yearbook of Agricultural Production, SIAP/SADER 2021 [5], the alfalfa production reported for the agricultural year 2020 (perennial cycle) (Table 1) corresponds to the total produced of green alfalfa, not including shrunken alfalfa production. Hidalgo occupies the second national place in alfalfa production (12.95%), only after Chihuahua (7,780,182.40 tons) with a production of 4,477,712 tons, for the same agricultural year (2021). Alfalfa generally is used as fodder, green or dried, in salads and flavored waters. The seed germinates from 2 or 3 °C, with an optimum temperature of 28 to 30 °C, and up to 38 °C, and can survive extreme droughts. It is rich in proteins, minerals, and vitamins [7].

It contains secondary metabolites such as saponins, coumarins, isoflavones, and alkaloids; their content differs with the type of cultivar, tissue, and stage of development. The aerial parts of alfalfa contain mainly glycosides of medicagenic acid substituted at C-3 by glucose or glucuronic acid, zanhic acid, and soyasaponin I tridesmoside [8,9]. It has been reported that the chemical composition of alfalfa sprouts (from the third day) presents water (869.1 g kg^−1^ DM), crude protein (68.2 g kg^−1^), ethereal extract (5.2 g kg^−1^), crude fiber (30.9 g kg^−1^), and ash (20.4 g kg^−1^). The composition of phytochemicals and bioactive compounds includes phytoestrogens, sterols, tocols, carotenoids, and saturated and unsaturated fatty acids. The main isoflavones found in alfalfa are secoisolariciresinol diglucoside, daidzein, secoisolariciresinol, coumestrol, isolariciresinol, hydroxymatairesinol, and matairesinol. The main sterols found in alfalfa are stigmasterol (1096.8 mg kg^−1^ DM), avenasterol (405.9 mg kg^−1^ DM), β-sitosterol (324.2 mg kg^−1^ DM), and campesterol (49.5 mg kg^−1^ DM). Important antioxidant compounds have also been identified: α-tocopherol (314.1 mg kg^−1^ DM), γ(β)-tocopherol (24.4 mg kg^−1^ DM), α-tocotrienol (4.1 mg kg^−1^ DM), δ-tocopherol (2.7 mg kg^−1^ DM), and γ-tocotrienol (2.1 mg kg^−1^ DM) [10].

Some reported uses for alfalfa seed flour (composition: 14.71% total starch, 37.59% crude protein, 3.74% ash, 26.22% total dietary fiber, 6.71% soluble dietary fiber, 19.51% insoluble dietary fiber) include improvement of the nutritional value of gluten-free biscuits using different levels of substitution for common rice flour (0, 15, 30, 45% *w*/*w*) [11]. In the same context of composition, some studies have reported the factors that may be responsible for variations in the composition and bioactivity of alfalfa (biotic and abiotic) and its biological effect by incorporation in diets. In addition to the inherent factors of plant growth conditions, the effect of light (LED), sound waves, drying, soaking, fermentation, and incorporation of selenium into the crop have been determined in alfalfa, which have diverse effects depending on the stage and potency of incorporation and/or application and the plant’s own metabolism. The incorporation of alfalfa in diets has improved the bioactive composition of the diets including polyunsaturated fatty acids (PUFA), isoflavones, tocopherols, anthocyanins, saponins and total polyphenols, with improvements in the antioxidant capacity of the diet, the consumption of some of these diets has improved the oxidative state of the *Longissimus dorsi* muscle of rabbits; also, an anti-cholesterol effect has been observed in chickens (see Table 2).

On the other hand, it has been reported [19] that alfalfa leaf peptides have a reducer power of 0.69 to 2.00 mg mL^−1^; they also presented 79.71% (1.60 mg mL^−1^) and 67.00% (0.90 mg mL^−1^) scavenging activity of the radicals DPPH (1,1-diphenyl-2-picrylhydrazyl) and superoxide, respectively. In addition, they chelated 65.15% of the ferrous ion at 0.50 mg mL^−1^. The molecular weight of 67.86% of the peptides was smaller than 1000 Da and was characterized by an amino acid profile with a high nutritional value (glutamic acid, aspartic acid, leucine, arginine, valine, lysine, among others). Alfalfa is a source of isoflavones such as genistein, daidzein, and glycitein [20], and tocopherols (tocols and tocotrienols), compounds with important antioxidant activity. The α-tocopherols are the most abundant of all the tocopherols; their biological activity is double that of the β and γ homologs and 100 times more than the δ homolog [21].

Saponins are a large group of compounds identified in alfalfa, consisting of nonpolar steroidal triterpenoids or aglycones (sapogenins) attached to one or more hydrophilic oligosaccharide moieties via ether or glycosidic ester bond [9]. Saponins function as a chemical protector in the plant’s defense system against harmful agents (e.g., pathogens), but bioactive effects such as antioxidants, antimicrobial, anti-inflammatory, antitumor, antidiabetic, anticholesterol, antiviral, immunomodulatory, antibacterial, antiparasitic, and allelopathic activity have also been identified. Due to their properties, saponins are used as natural surfactants in foods, antimicrobial preservatives, and natural emulsifiers [8,9]. The structural differences between saponins have an impact on the bioactivity demonstrated; for example, due to the lack of sugar, sapogenins have shown better chemical properties (lower molecular weight, higher lipophilicity, or lower molecular flexibility) that improve permeability and bioactivity, in comparison with the precursor saponin [9].

In addition, polysaccharides (e.g., hemicellulose and pectin) with important bioactive functions, such as antioxidant, antitumor, immunomodulatory, anti-inflammatory, and growth-promoting properties, have been obtained from fresh alfalfa [22,23,24]; they have also been recognized as natural alternatives to antibiotics when added to animal diets. A pectic polysaccharide from the alfalfa stem was identified as rhamnogalacturonan I (RG-I; pm 2.38 × 10^3^ kDa) [22]. The polysaccharide (50 µg mL^−1^) showed a significant anti-inflammatory effect against mRNA expression of the pro-inflammatory genes of the cytokines interleukin (IL)-1β and IL-6, which suggests a potential use in functional foods and supplemented products. Another studied polysaccharide of alfalfa is that formed by galacturonic acid (146.500 μg mg^−1^), glucose (39.092 μg mg^−1^), glucuronic acid (29.343 μg mg^−1^), arabinose (12.282 μg mg^−1^), galactose (8.649 μg mg^−1^), mannose (6.791 μg mg^−1^), xylose (4.811 μg mg^−1^), and fucose (4.346 μg mg^−1^). In vitro studies showed that 50 and 100 µg mL^−1^ of the polysaccharide increased the cell viability of macrophages (RAW 264.7) by improving their immune functions, as well as the secretion and gene expression of inflammatory factors (cytokines, NO/iNOS, IL-6, and tumor necrosis factor (TNF)-α) [23]. The same research group reported a characterization for this same polysaccharide, indicating that the molar ratio of the saccharides is 2.6:8.0:4.7:21.3:3.2:1.0:74.2:14.9 for fucose, arabinose, galactose, glucose, xylose, mannose, galacturonic acid, and glucuronic acid, respectively [24]. Furthermore, the polysaccharide markedly increased the proliferation of B cells and the secretion of IgM in a dose- and time-dependent manner but not the proliferation and expression of cytokines (IL-2, -4, and IFN-γ) of T cells. This represents a biological activity that contributes to the immune system [23]. For alfalfa polysaccharides, in a mouse embryonic fibroblast (MEF) model with oxidative stress induced by hydrogen peroxide (150 µM; H_2_O_2_), the activation of antioxidant capacity (1.0 mM g^−1^ (T-AOC)) was detected as a preventive defense mechanism; and 250 µM/12 h was considered as the optimal concentration to stimulate stress in MEF (because it presents the highest expression of the pro-inflammatory gene related to senescence RIG-I). A concentration of 20 µg mL^−1^ of polysaccharides exhibited the greatest antioxidant effect and the least secretion of inflammatory cytokines [25]. The results demonstrate that alfalfa polysaccharides exert a protective action against oxidative damage induced by hydrogen peroxide.

Another study conducted to determine the effect of alfalfa (*Medicago sativa* L.) polysaccharides considered the growth performance and intestinal health of 200 piglets (35 days old) [26]. Biologically active phytogenic polysaccharides mainly contain carbohydrates comprising β-1,3-D-glycan units. Supplementation with the polysaccharide (0, 300, 500, 800, or 1200 mg polysaccharide kg^−1^ diet for 42 days) increased average daily gain (ADG) and feed ratio (G/F) in a dose-response manner. The experimental group receiving 500 mg kg^−1^ of polysaccharide in the diet showed the highest *Lactobacillus* values in the cecum, colon, and rectum. And the values for *Salmonella* and *Escherichia coli* decreased in all sections of the large intestine. The results showed that supplementation of the diet with alfalfa polysaccharides (500 mg kg^−1^) improved intestinal morphological development and amylase and protease activity in the small intestine and promoted beneficial microbial populations in the large intestine [26].

It determined that alfalfa fiber (12 and 18% in the diet of piglets) decreased diarrhea and increased the composition and diversity of fecal bacteria (*Bacteroidetes* and *Firmicutes* were the dominant phyla (98% of the total)), and consequently improved the growth performance of weaning piglets [27]. The supplementation of alfalfa fiber (6–12%) in the diet of 48 crossbred piglets significantly increased growth performance and crude protein digestibility [28], particularly that of albumin, globulins, and total protein; however, it decreased levels of glucose (6% supplemented fiber, from 3.87 to 3.75 mmol L^−1^), cholesterol (12% supplemented fiber, from 2.3 to 2.06 mmol L^−1^), triglycerides (12% supplemented fiber, from 0.60 to 0.47 mmol L^−1^), aspartate aminotransferase (6% supplemented fiber, 48 to 46 µ L^−1^), and alanine aminotransferase (6% supplemented fiber, 42.5 to 39.5 µ L^−1^).

### 2.2. Maguey (Agave *spp.*)

According to Statistical Yearbook of Agricultural Production, SIAP/SADER 2021 [5], the maguey production reported for the agricultural year 2021 (perennial cycle) (Table 1), corresponds to the total produced of pulquero maguey of aguamiel (58.28% national), not including unclassified pulquero maguey production. Hidalgo occupies the first national place in maguey production, followed by Tlaxcala and Mexico. The same statistical record does not report agave production for the state of Hidalgo, thus recognizing a difference in the use of the terms (maguey and agave) concerning the species and products produced from the plant: the *Agave salmiana* also named pulquero maguey to produce pulque, and *Agave tequilero* known to produce tequila.

Maguey, also called mixiotero magueys (*Agave salmiana*) is a plant with rosette leaves, thick and fleshy, with a short stem, and a lower pineapple that does not protrude from the ground. In Mexico there are about 200 species of maguey, a term applied to species of the genus *Agave* (Asparagaceae). It requires low-humidity soil, intense light, temperatures of 15 to 25 °C, and an approximate altitude of 1700 to 2400 m above sea level [7]. Species of the genus present an important profile of phenolic compounds, such as flavonoids, homoisoflavonoids, and phenolic acids, which have been widely related to important biological, antioxidant, antibacterial, antifungal, antinematode, and immunomodulatory activity [29]. In addition, species of the genus *Agave* are recognized as an important source of monosaccharides as fructose to produce traditional alcoholic beverages, natural fibers, saponins, high-fructose syrups, and fructans; even the different phytocomponents of the thick leaves act as seasoning or flavor sources during the roasting of meat to prepare a barbecue [30,31]. As a source of saponins, the use of agave is emphasized as having antibacterial, antientomological, antifungal, anticholesterolemic, and anticancer effects) [32]. Agave also contains policosanols and sapogenins; agamenone (5,7-dihydroxy-6,5′-dimethoxy-3′,4′-methylenedioxy flavanone), flavonol, or isoflavones have been identified in concentrated honey water [33]. Mature plants contain low concentrations of saponins, and silage reduces their quantity, improving their characteristics for livestock feed [31].

About 30% of the agave plant is made up of leaves, which have few applications. Saponins are mainly present in the leaves and can be used as precursors for sterols of therapeutic importance. Leaves of *A. salmiana* and *A. tequilana* Weber were structurally characterized (light microscopy) and methanolic extracts followed by dichloromethane were recovered, where the presence of saponins was confirmed by hemolytic activity in erythrocytes and a positive reaction with anisaldehyde reagent. *A. salmiana* presented a higher percentage of protein (7.3%) [34,35].

Multiple in vivo tests have been reported to demonstrate the diverse bioactivity of *Agave* spp., not having many outstanding investigations on *A. salmiana*. *A. salmiana* syrup (honey water) is reported to have antioxidant activity of up to 1096.8 µM TE by DPPH, and a total phenolic content of 904.8 µM GAE [31]. The antidiabetic activity of high-fructose agave syrup from *A. salmiana* protected against liver steatosis in rats fed 2 and 5 g of serum kg^−1^ and had a quadratic opposite effect on glycosylated hemoglobin in the blood of diabetic rats (dose 0.5 g kg^−1^) [36]. Some patents [33,37] reported anticancer activity for the methanolic extract (80%) of concentrated agave syrup (10%) from the species *A. atrovirens*, *A salmiana*, and *A. lehmanni* (15 mg mL^−1^), with 84.89% inhibition of colon cancer cells (Caco-2) and 67.95% of liver cancer cells (HepG2). In addition, antioxidant activity for the same extract was reported of 61.87 µmol ET g^−1^ sample. These properties are attributed to the composition that includes phytosterols, polyphenols, flavonoids (agamenone), tannins, policosanols, inulin, and saponins (sapogenins).

Another study was carried out on sap concentrated from *A. salmiana*, evaluating the apoptotic activity in HT-29 cells (IC_50_, of 3.8 mg mL^−1^ for the concentrate) of saponins from the acetonic extract. The most bioactive fractions (up to 80% cell inhibition at 75 μg mL^−1^) presented an IC_50_ of 108.4, 82.7, and >250 mg mL^−1^, respectively (partition coefficients (Kd) of 0.23, 0.33 and 0.40); they contained steroidal saponins, mainly magueyoside B (266.4 μg PE mg^−1^; Kd of 0.33). Flow cytometric analysis has determined that the fraction rich in the glycosides kammogenin and manogenin induces apoptosis and that the presence of gentrogenin and hecogenin is related to a necrotic effect [38]. The phytochemical composition has been proposed as a tool for the classification of different agave syrups (*A. tequilana* (>60% fructose) and *A. salmiana* (sucrose 28–32%)), such as infrared spectroscopy coupled to chemometrics (NIR-MIR-SIMCA-PCA) and high-performance anion exchange chromatography with pulsed amperometric detection (HPAEC-PAD) [39]. The said techniques are reported to identify and classify without significant mistakes. ^1^H-NMR-spectroscopy-PCA was used to characterize syrup profiles and chemometrics, what allowed the sweeteners’ classification by origin and kind of Agave. The agave syrups exhibited appreciable amounts of saponins, cardiac glucosides, and terpenoids (excelled in color intensity in the reaction) followed by glucosides, quinones, flavonoids, and coumarins in moderate amounts. In *A. tequilana* syrup, flavonoids and terpenoids were only detected in a few samples. *A. salmiana* syrup displayed a positive colorimetric reaction for all the evaluated compounds [40].

In addition to the apoptotic activity, it has been reported [41] that antioxidant activity of agave sap (*A. salmiana*) is dependent on storage time and is correlated with the browning developed due to heating and storage time, increasing from 18 to 23 eq Trolox µmol g^−1^ DW in a lot with a high degree of browning (57.7 OD_490_ g^−1^ fw), after 20 weeks of storage. In addition, they reported that the content of saponins (kammogenin glycosides (magueyosides A and B), manogenins, and hecogenin (agavoside C′)) was different per batch, varying from 224.2 to 434.7 PE g^−1^ DW in week 2 and varying up to week 20 of storage (207.7 to 462.4 PE g^−1^ DW). They found no correlation of the browning index and antioxidant capacity (ORAC) with the concentration of free amino acids (serine, phenylalanine, and lysine); the positive correlation found was browning with furosine, an early derivative of the Maillard reaction of lysine, reported as a free radical scavenger [41]. The presence of several compounds in agave syrup (ethanolic extracts), such as saponins, flavonoids, quinones, glycosides, cardiac glycosides, terpenoids, and coumarins, has been identified for several species [40]. The antioxidant activity of agave syrups (*A. tequilana*, *A. salmiana*) was in the range of 10–53%, while the content of total phenols was from 24 to 300 GAE 100 g^−1^ and that of condensed tannins was from 240 to 1900 mg CE g^−1^. In addition, a relationship between the color and the antioxidant capacity of the syrups is reported, with dark syrups such as those of *A. salmiana* having the highest antioxidant capacity, about 28.33%, while light syrups show an average capacity of 8.7%. In tests on mice, the consumption of fresh and boiled syrup (*A. salmiana* sap) promoted weight gain (13%) and increased hemoglobin counts to 4.5 and 9%, respectively; the hematocrit count increased from 2.6 to 5.3%; iron, transferrin, ferritin, and phosphorus increase with the consumption of fresh syrup, while iron increase with boiled syrup [42]. The antioxidant capacity of syrup was determined as 7.1 μmol GAE g^−1^ DW, that for commercial coffee being 156.1 μmol GAE g^−1^ DW (commercial coffee). No adverse effects of syrup consumption it is observed.

Due to the importance of maguey, *A. salmiana*, in the production of pulque and functional food industries, processes have been sought to maintain its production and take advantage of its benefits (Table 3). The micropropagation of agave can be an auxiliary process to increase the phytochemicals and bioactivities in the plant. The increase in antioxidant activity in micropropaged plants in vitro has several possible causes: the presence and interaction of cytokines and auxins [43], or the highest concentration of phenols or saponins [44], or bioactive compounds in general, which can be responsive to culture conditions, such as micropropagation [45], which has already been shown to have diverse effects on compositions and properties. Micropropagation needs more research (at different conditions, ages, and physiological states), together with scaling up to primary production levels, so that the effects found in micropropagation can be exploited in the primary production sector and from there to the industrial sector. The study of technologies for the extraction of bioactives from agave bagasse is little to date, the effect of ultrasound assisted with supercritical fluids in *Agave salmiana* has reported antioxidant activity and saponin content [46], which is a promising area for the recovery of bioactives and the valorization of agave processing residues (Table 3).

Antioxidant properties and bioactive compounds of methanolic extracts of *A. salmiana* leaves were evaluated [50] at different stages of development (I–VI, from 1 to 7 years). The total phenolic content from leaves extracts was found to be between 5 (stage VI) and 13 mg GAE g^−1^ (stage II) the maximum; the antioxidant capacity presented a negative trend from stages I to VI (from 146 to 52 μmol TE g^−1^ respectively), the flavonols showing the same behavior (65% reduction from stages I to VI). Five saponins were identified (chlorogenin glycoside 2, chlorogenin glycoside 1, hecogenin glycoside 1, tigogenin glycoside, hecogenin glycoside 2) (also reported [48] in addition to flavonols (maximum concentrations in stage I, kaempferol (0.045 mg g^−1^ DW) and quercetin (0.07 mg g^−1^ DW)). Stage III and IV plants presented the highest content of saponins, mainly chlorogenin glycoside, at 3.19 and 2.90 mg PE g^−1^, respectively. According to Pearson’s correlation, there is a positive relationship between total phenol content and antioxidant activity. Based on these results, it can be said that *A. salmiana* plants from stages I to IV could be a good source of antioxidants and bioactive agents; in addition to that, the concentration of metabolites could be a marker of the developmental stage. In the same way, the content of saponins was evaluated [51], regarding maturity (before (immature) and at the beginning of the reproductive stage (mature)) of the agaves (8 years old) and syrup. Saponins derived from kammogenin, manogenin, gentrogenin, and hecogenin were found. In syrup form immature *A. salmiana*, the saponin content was twice as high (478.4 μg PE g^−1^ DW of syrup) as that of immature *A. americana* (179.0 μg PE g^−1^ DW of mead). For both species, the saponin content decreased when the plants reached sexual maturity (up to 325.7 and 60.5 μg g^−1^ DW of syrup, for *A. salmiana* and *A. americana*, respectively). This finding is important to better select the species and maturity stage of agaves used as a source of bioactive.

In addition to research on stems and syrup, the agave flower has also been studied. The traditional use of the *A. salmiana* flower as an anthelmintic agent, insecticide, and antimicrobial occurs in some regions of Mexico. The antibacterial activity against Gram-negative bacteria of the flower scape was demonstrated [52], these being more susceptible to aqueous and crude extracts than Gram-positive bacteria. The aqueous extract presented better yields (5.935 g 100 g^−1^ DW; young stage of development, upper section), antioxidant activity (78.55% by DPPH; young stage of development, middle part), and total phenolic content (199.12 mg GAE 100 g^−1^ of extract; young stage of development, middle part); the highest antimicrobial activity was found for the aqueous extract of the mid-development and mid-section sample (vs. *E. coli*, *Salmonella typhimurium*, and *Shigella sonnei*) due to the possible presence of saponins, tannins, flavonoids, terpenes, and alkaloids on the flower scape of agave. The best minimum inhibitory concentration (MIC; 6.83 mg mL^−1^) was found for the ethanolic extract of the adult stage and middle part of the flower, against *Shigella sonnei*.

An important aspect in the extraction of bioactive compounds from underutilized leaves of *Agave* spp. is the structural characteristics of these leaves. A study was reported of structure, water permeability, and resistance to sterilization of the mixiotera leaves or cuticles of agave [53]. The cuticle of *A. salmiana* is a material that resists humid heat when sterilized under pressure conditions (121 °C and 15 lb in^−2^, 15 min); it has a porous structure that linearly regulates the diffusion of water. The cuticle (mixiote) consists of a lipid matrix based on cutin (40–80%) and is also made up of fatty acids (C16:18) linked by ester-type bonds. In addition, it has been reported that agave leaves are an important source of calcium, particularly *A. salmiana*, one of the species that does not present oxalate crystals; they have been identified in *A. atrovirens*.

Another type of important compound in agaves is fructans, which are the main photosynthetic product in *Agave* spp.; their main function is to store energy and act as an osmoprotectant during periods of drought. Fructans (fructooligosaccharides or inulin) are of nutraceutical interest due to their resistance to human digestion and subsequent fermentation by the colonic microflora, producing short-chain fatty acids [31]. Agave (*A. salmiana*) fructans are involved in the activation and selective differentiation of cells (peripheral blood mononuclear cells (PBMC)) of the immune system (immunomodulator) through interactions with probiotics (*Lactobacillus casei* and *Bifidobacterium lactis*). The agave fructans showed the highest prebiotic activity and increased levels of CD69 expression, proliferative activity and NO production when administered with the probiotic *L. casei* [54]. Short- and medium-chain branched fructans from agave have been tested as prebiotics and have been shown to increase lactobacilli in a dynamic in vitro model of the large intestine. With both substrates, butyrate production was increased, which may have a beneficial impact on health, and the production of potentially harmful putrefaction metabolites was reduced. Based on these findings, agave fructans should be considered as potential prebiotics [55].

Fructans (with β (2→6) linkages; from *Agave tequilana* blue variety) have been shown to attenuate the production of proinflammatory cytokines from stimulated dendritic cells and strongly inhibit Toll-like receptors, thus exerting an anti-inflammatory effect. The structure of fructans should be carefully determined and taken into consideration when intended to be used as food supplement, as the presence of linear or branched structure, the chain-length, as well as the dose of these molecules can exert differential responses. Further studies are needed to establish specifically in which disease state agave fructans could serve as an alternative or supplemental therapeutic option [56]. The exact mechanisms of effect of agave fructans are still under investigation for a better understanding of the benefits to human health and technological developments, for example, in the interactions with the intestinal microbiota that benefit the growth of beneficial bacteria and reduce disorders with glucose metabolism, fats, metabolic syndrome, calcium absorption, mental disorders, oxidative stress and cancer. The properties of agave fructans depend on various structural factors and management conditions, so research on them is a promising and long-term line [57].

### 2.3. Maize (Zea mays *L.*)

According to the Statistical Yearbook of Agricultural Production, SIAP/SADER 2021 [5], the corn production reported for the agricultural year 2021 (agricultural year) (Table 1), corresponds to white grain corn (617,696.87 tons) plus a portion of yellow (459.98 tons) reaching 618,156.85 tons in total (2.25% national); and green fodder corn with 130,136.92 tons (0.75% national), without presenting the production of shrunken or dry fodder corn. Hidalgo ranks thirteenth in national grain corn production and tenth in national production of green forage corn.

Corn belongs to the kingdom *Plantae*, division *Magnoliophyta*, class *Liliopsida*, subclass *Commelinidae*, order *Poales*, family *Poaceae* (Gramíneae), subfamily *Panicoideae*, genera *Zea* and specie *Z. mays* (https://www.biodiversitylibrary.org/page/358992#page/1/mode/1up (accessed on 12 August 2022)); in Mexico, pre-Hispanic cultures called the ear centli and the grain tlaolli [58]. For its development, grain corn prefers loamy-loamy, loamy-clayey, and loamy-clayey-silty soils with a depth greater than or equal to 1 m, with a pH of 5.5 to 7.5, at an optimum temperature of 18 to 24 °C and requires an average annual rainfall of 700 to 1300 mm [7]. Some of the products made from the transformation of corn are starch, liquid sugar, rectified alcohol, germ, gluten, feed, and bioethanol, in addition to some products called “biorefined” such as sweeteners, polysaccharides, pharmaceuticals, nutraceuticals, fibers, biodegradable films, organic acids, pigments, polyols, and vitamins, among others [59]. In Mexico the products derived from corn that stand out are tortillas, forage, oils, biofuels, starches, glucose, fructose, dextrose, ethanol, atole, and tesquiño [58]. The corn breeds that are grown in Hidalgo include the yellow rice, the cacahuacintle, the chalqueño, the conical and conical norteño, the conical elotes, the mushito, and the negrito, according to the National Commission for the Knowledge and Use of Biodiversity (CONABIO) (https://conabio.shinyapps.io/conabio-pgmaices1/ (accessed on 12 June 2022)). Although, as mentioned before, the statistical data from SADER mention the varieties: white grain corn, yellow grain corn, and green fodder corn.

The composition of blue and white corn flours (g 100 g^−1^) has been reported [60] as follows: moisture 9.8 and 7.0, protein 9.1 and 8.4, lipids 5.2 and 4.7, ash 1.1 and 1.3, total starch 70.7 and 74.2, total dietary fiber 10.9 and 11.2, and soluble carbohydrates 3.0 and 0.3, respectively. Total carbohydrates represent the majority fraction in purple corn, these being highly available. Starch is the largest component in corn grains (63.48–89.9 g 100 g^−1^ DW), with amylose of 20.70–33.32 g 100 g^−1^ of starch; other components are proteins (6.73–11.37 g 100 g^−1^ DW), lipids (1.80–7.53 g 100 g^−1^ DW), and ash (1.40–2.06 g 100 g^−1^ DW) [61]. The composition of purple corn reported [62] presents humidity (10 g 100 g^−1^), ash (1.71 g 100 g^−1^), protein (9.10 g 100 g^−1^), fat (1.80 g 100 g^−1^), fiber (11.20 g 100 g^−1^), starch (57.70 g 100 g^−1^), amylose (27.10 g 100 g^−1^). Purple corn has been found to have a lower starch content and a lower glycemic index than other types, such as white corn. The parts of corn differ in composition: the pericarp has high fiber content, the endosperm is rich in starch, and the germ contains proteins, fats, sugars, and ash [58]. The bioactive compound that has been determined as predominant depends on the portion of the grain; for example, anthocyanins have been in the pericarp, aleurone [58,63], and endosperm; and phenolic acids in the pericarp [58].

Mexican corn in its different varieties has shown antioxidant activity, quinone reductase induction, antimutagenic activity (*S. typhimurium* TA98), and antidiabetic activity. Of course, all properties depend on the type of grain, its physiological development, the portion of grain evaluated, the extraction technique used (e.g., solvents), the previous handling of the grain (storage, pretreatment) and the physicochemical properties of the compound to be evaluated, among others. Intense colored grains (purple, blue) stand out in bioactive composition, as well as in their bioactivities. In addition, it has been reported that some processes can modify the composition of compounds and therefore their bioactivities, for example, nixtamalization reduces the antioxidant capacity of ethanolic extracts of white, red, blue, and purple corn, but increases the relative percentage of glycosylated anthocyanins and decreases acylated anthocyanins in raw blue corn grain. On the contrary, air drying of grains did not have a significant effect on desorption isotherms of several varieties (Spanish, white, yellow, yellow, purple). Similarly, cooking in water resulted in an increase in the total phenolic content and antioxidant capacity of blue corn. Loss of anthocyanin content has been observed (83%) in the mass of nixtamalized blue grains and in tortilla processing (64%) [64]. For white grains, nixtamalization also reduced carotenoids by 53 to 56%, but not antioxidant activity and antimutagenicity. Despite the losses in concentration (the blue variety standing out for anthocyanins and the red variety for carotenoids), the pigments of creole corn showed antioxidant and antimutagenic activity after nixtamalization. For details see Table 4.

Corn in its various varieties presents in its composition polyphenols (catechin and epicatechins linked to acylated and non-acylated anthocyanins) [58,73,75,76,77], flavonoids (naringenin, kaempferol, rutin, morin, and quercetin and hesperidin derivatives), phenolic acids (protocatechic, vanillic, p-coumaric, m-coumaric, o-coumaric, chlorogenic, caffeic, rutin, ferulic, hydroxybenzoic, and sinapic acids, and hydroxycinnamic acid derivatives) [58,78,79,80], carotenoids, and anthocyanins, compounds whose concentration depends on the coloration [59,72,81]. The average percentages of anthocyanins in blue corn are 90% cyanidin, 8% pelargonidin, and about 2% peonidin [58]. Compounds such as cyanidin-3-glucoside, pelargonidin-3-glucoside, peonidin-3-glucoside, cyanidin-3-(maloyl)-glucoside, pelargonidin-3-(maloyl)-glucoside, and peonidin-3-(maloyl)-glucoside, among others, have been detected in powdered purple corn cultivars [68,77,79,82,83]. Of the total anthocyanins, cyanidin-3-glucoside has been reported as the main anthocyanin in red (51%), blue (49%), multicolored (47%), and purple varieties (31%), while pelargonidin-3-glucoside (43%) predominates in those with pink coloration [61]. It has been detected that the accumulation of anthocyanins in corn leaves can be induced by the application of organic selenium (selenomethionine) to the plant; this could be a symptom of selenium phytotoxicity or indicate a change in the oxidative state of the plant. In food, the presence of anthocyanins is desirable for their nutraceutical and antioxidant properties [18]. The chemical form in which anthocyanins are found has also been reported to exert an effect on the anticancer capacity; thus, the acidified form has the better capacity, at least against the colon (Caco-2), liver (HepG2), breast (MCF7), and prostate (PC3) cancer cells [76].

The seeds and the cob of purple corn have shown antioxidant activity [82], which is affected by the maturation stage (grain) and type of corn, for the reported techniques [78]. It has also been reported that the composition of total polyphenols and anthocyanins (flavonoids) of purple corn powder (cob) cultivars depends on the sowing area, but not on the sowing density or type of fertilizer used [77]. Additionally, it has been seen that the concentration of anthocyanins depends on the degree of maturity of the grains, immature corn being proposed as a better source (67.1–88.2% acylated anthocyanins (with acylated radical) in young plants and 46.2–83.6% at the mature stage). The antioxidant capacity increases with maturation (genotype KKU-WX111031) as determined by the DPPH technique (from 11.7 to 21.6 µmol TE g^−1^ DW), by FRAP iron-reduction techniques (from 69.8 to 159.2 µmol Fe (II) g^−1^ DW), and by Trolox equivalents (from 95.4 to 156.8 µmol TE g^−1^ DW) [83].

In general, it is reported that the concentrations of compounds with antioxidant capacity in dark purple corn kernels are less affected by the application of various soaking and heat treatments; the opposite effect was determined for pearling, which suggests that those compounds are concentrated in the husk of the grain. Purple corn presents higher concentrations and better antioxidant capacity than the reddish variety (Sangre de Cristo) [84]. The total phenolic content and antioxidant capacity of cooked blue corn flours have been reported to be higher than for uncooked flours, so heat treatments appear to cause such behavior. Blue corn flour has a higher resistant starch content and slow digestion; therefore, it exhibits a lower glycemic index (63) than white corn (71) (without cooking). Cooked flour of both varieties has a higher glycemic index, 78 and 82, respectively; in the case of cooked flour of blue corn the interaction with polyphenols, it could be responsible for the decrease of glycemic index, due to the low digestibility of the complexes formed. An analysis of correlations indicates that the increase in polyphenols causes a decrease in the predicted glycemic index in model systems of blue and white corn [60]. Regarding in vivo tests [85], dehydrated and micro pulverized purple corn was administered in 1 g capsules (2 g daily for 30 days) to a group of diabetic patients with mixed dyslipidemia, without previous treatment. They observed better fasting glycemic control (from 211.3 to 112.5 mg dL^−1^), obtaining a 46.8% reduction from baseline levels. Additionally, purple corn lowers triglycerides and increases HDL cholesterol according to this research.

Purple corn extracts have been proposed as low-cost colorants, as they are concentrates of anthocyanins [86], and as food antioxidant additives, for example in mayonnaise, where the extract (0.4 g kg^−1^) presented better storage performance than commercial antioxidants (BHT and EDTA) [87].

### 2.4. Forage Oats (Avena sativa *L.*)

According to the Statistical Yearbook of Agricultural Production, SIAP/SADER 2021 [5], the oat production reported for the agricultural year 2021 (agricultural year) (Table 1) corresponds to green forage oats without classifying (no variety determined), excluding production of shrunken or dried forage oats. Hidalgo ranks ninth in the country (3.24%) in green forage oat production. The grain oats produced in the state of Hidalgo are 2709.49 tons, a quantity much lower than the forage (324,378.96 tons) for the same agricultural year. Oats are used mainly in livestock feed, as a forage plant, in the pasture, and as silage. The oat is an annual herbaceous plant, of the grass family. It is from cold climates and is very sensitive to high temperatures, mainly during flowering and grain formation. It requires a lot of water for its development because it presents great transpiration; it grows better in deep, clay-sandy soils, rich in lime but without excess, and that retain moisture. Oats are highly adapted to acidic soils, which is why they are usually sown in soils rich in organic matter [7].

It is commonly known that oats (mainly *Avena sativa* L.) affect satiety and delay the absorption of nutrients, as well as having a deterrent action against various disorders of the gastrointestinal tract. The effects are mainly attributed to the soluble fiber content [88]. Oatmeal as a functional food has physiological benefits in reducing hyperglycemia, hyperinsulinemia, hypercholesterolemia, hypertension, and cancer; for these actions, the β-glucans of oats are considered beneficial in the prevention, treatment, and control of diabetes and cardiovascular diseases (Table 5).

Other bioactive substances that contribute to the medicinal action reported for *Avena sativa* L. are polyphenols (>20), phenolic acids, alkyl resorcinols, and avenanthramides [99,100,101] that exhibit antioxidant, anti-inflammatory, and antiproliferative activity, which inhibits cancer cell progression [99,102]. Phytosterols in a mixture with β-glucan have shown strong anti-cholesterol properties (low-density lipoprotein (LDL) and total cholesterol). Epidemiological data and clinical trials suggest that a 0.026 mmol L^−1^ increase in LDL cholesterol causes a 1% increase in coronary risk [88] and that for every 1% lowering of serum cholesterol levels, the risk of developing coronary heart disease is reduced by 2–3% and reducing insulin levels reduces the risk of developing insulin insensitivity and metabolic syndrome. Oat bran reduces total serum cholesterol in hypercholesterolemic subjects by as much as 23% with no change in HDL cholesterol [103].

A high fiber intake can improve the conditions of the intestinal environment by diluting carcinogens in the colon and decreasing transfer time. The oat bran β-glucans can regulate glucose metabolism, reducing hyperglycemia, especially in high doses (2000 mg kg^−1^) like metformin, in diabetic rats and in vitro tests. A dose of β-glucans has fewer adverse effects than traditional therapy for diabetes mellitus and its intake is approved by the Food and Drug Administration (FDA) at 3 g d^−1^, for nutritional and bioactive effects [92]; dose equal to that concluded by a meta-analysis of 30 research articles on the effect of different levels of exposure to β-glucans reported a dose-response model [94]. The β-glucans in oats are (1,3) or (1,4)-β-D-glucans linear polysaccharides, mainly composed of (1,3) units of cellotriosyl and cellotetraosyl (>90%) [88]. To the mixed-linkage (1,3), (1,4)-β-D-glucans (β-glucans) present in cereals at 30 and 70%, respectively, are attributed a significant number of functionalities and roles that make them unique as part of the plant cell wall and as soluble dietary fiber [103]. The (1,3) bond prevents tight packing of the molecule and causes its partial water solubility. The characteristic molar ratio reported for tri/tetra-oligosaccharides is 2.1–2.4 [88]. Glucans (1,3 and 1,4) from oats are highly soluble in water, have low viscosity and strong biological activity, are part of the human diet, and can contact enterocytes, immune cells, and dendritic cells, that are present in the intestinal immune system and can increase its function [96]. Oat β-glucans are a good agent for reducing total (for each 3 g of dietary fiber per day reduces total cholesterol ≈ 2%; for each 2.9 g of β-glucan twice a day reduces the levels of total cholesterol 9.2%) and LDL cholesterol (for each 2.9 g of β-glucan twice a day reduces the LDL cholesterol 10%), improve HDL cholesterol, are a good regulator of blood pressure, improve the lipid profile of the blood, are regulators of postprandial blood glucose (for each gram of β-glucan consumed, the glycemic index decreases by 4 units) and insulin response, and reduce and maintain body weight. Therefore, they help to treat and/or prevent cardiovascular diseases and diabetes; improve immune functions by increasing immunoglobulins, NK cells (natural killer), and killer T cells (lymphocytes) in the blood; they improve resistance to infectious and parasitic diseases; and contribute to a reduction in the risk of cancer and to improve the quality of chemotherapy. The different effects of ingesting β-glucans are due to the type of source, whether they are grains or extracts, frequency, dose, and molecular weight, as well as the age, gender, physiology, and initial levels in individuals [88,103].

The viscosity of β-glucan (in drink mode) could account for 79–96% of the changes in the plasma response of glucose and insulin to 50 g of glucose [103], delaying intestinal glucose absorption as it is trapped in micelles with β-glucan, attenuating the postprandial insulin response (in portal vein), and consequently decreasing the activity of the liver enzyme HMGCoA; these phenomena have beneficial effects in the control and prevention of type 2 diabetes and cholesterol synthesis [88,91]. Another possible mechanism is reducing pancreatic amylase activity and reducing the movement of sugars released into the intestinal wall. It has been identified that a high viscosity of β-glucan extracts (in an in vitro digestive system), impacts on reducing the digestibility of starch, in addition to reducing the absorption of glucose in the blood; this is better presented with 40 g of starch in the formulation, than with 60 g [93]. The intake of purified oat β-glucans (in pigs) reduced the net glucose absorption, which impacts the reduction of the insulin delivery peak (6% β-glucan, 30 min) while maintaining pre-hepatic insulin homeostasis [91]. The cause of the decrease in glucose absorption is reported to be an increase in the water-binding capacity and the viscosity of the gastrointestinal contents, in addition to a possible effect due to gastric emptying. The effect of an increase in the viscosity of the food bolus, from the upper part of the gastrointestinal tract, lengthens gastric emptying, motility, and residence times, and the absorption of nutrients, which is subsequently reflected in a decrease in blood glucose and insulin [88]. Mealtime intake of β-glucans (8.9 g d^−1^) has been observed to result in carbohydrate- and lipid-like metabolisms, decreasing postprandial glucose and delaying and/or reducing carbohydrate absorption in the intestine [104]. The inhibitory effect of viscous soluble fiber on the postprandial increase in glucose and insulin is decreased when the viscosity of the prepared fiber is reduced by acid treatments [90].

Similar mechanisms of β-glucans are reported to decrease cholesterol absorption [88]: the increase in viscosity reduces the available lipids, slows down diffusion, and modifies the thickness of the non-agitated layer at the intestinal absorption site; entrapment in whole micelles containing bile acid, in the intestine, avoids interaction of the lipid with the luminal membrane transporters in the intestinal epithelium and decreases fat emulsion in the small intestine, actions that increase the excretion of bile acids in feces of 35–65%. The low bile acid, the hepatic conversion of cholesterol to bile acid increases, hepatic cholesterol stores decrease, and, to reach a steady-state, endogenous cholesterol synthesis increases. The restoration of hepatic cholesterol produces a decrease in serum LDL cholesterol. Intestinal absorption of lipids [90] was evaluated in vitro in the presence of β-glucans from oats and barley (0.1–0.5% *w*/*w*) in an intestinal cell line (NCI-H716) in rats. A decrease in the absorption of fatty acids (18:0, 18:2) was detected due to the effect of β-glucans; this effect varies according to the source of β-glucans, the presence of resistance (aqueous layer, viscosity, agitation), and the portion of intestine evaluated, in addition to the inhibition of regulatory genes for intestinal absorption and lipid synthesis (FAS, ACC, SREBP-1a, SREBP-1c, SREBP-2, i-FABP, FATP4 mRNA), and to the possible participation in the metabolism of enterocytes or transmembrane transport mechanisms. The enzymatic hydrolysis of oat glucans (730,000 g mol^−1^) improved anticholesterolemic activity, specifically reduced rat serum triglycerides and weight gain, increased HDL cholesterol, and reduced cholesterol. For some tests, there was no significant difference in the effects of hydrolyzed and native β-glucan on LDL [89].

β-glucans may be one of the types of compounds responsible for the immunological effects (innate and adaptive) provided by cereals, fungi, algae, yeasts, and bacteria [96]. The immunomodulatory effect is due to the binding of β-glucans to immune receptors (dectin-1, receptor 3 (CR3; CD11b/CD18), lactosylceramide) that promote a group of immune cells such as monocytes, macrophages, neutrophils, NK cells, and tooth cells. Binding to receptors also promotes the release of cytokines such as IL-12, IL-6, IL-10, and TNF. The antitumor efficacy of (1,3)-β-glucan is related to the type of tumor, the genetic background of the host, the dose, and the route and moment of administration of the glucan, as well as the tumor burden. Proposed mechanisms of anticancer activity include the destruction of tumor cells by macrophages, and modulation of the activity of lymphocytes, neutrophils, and NK cells; in these cases, the action of β-glucans in combination with immune mechanisms is proposed [88]. The antitumor activity of high- and low-molecular-weight oat β-glucans was evaluated in two human lung cancer cell lines (A549 and H69AR) and normal keratinocytes (HaCaT) [98]. High-molecular-weight β-glucans from oats did not show significant toxicity to normal cells but did cause a decrease in cancer cell viability (about a 50% decrease at 200 µg mL^−1^). The oxidation marker malondialdehyde was increased in both cancer cell lines, indicating a possible induction of oxidative stress by the presence of β-glucan; the high expression of mitochondrial superoxide dismutase and significant changes in the cytoskeleton of cells confirm the hypothesis [88]. In addition, the low-molecular-weight β-glucans from oats significantly decrease the viability of cancer cells with increasing concentration (400 μg mL^−1^) and incubation time [96]. The mechanism by which β-glucans can kill cancer cells is very complex and not fully understood.

Avenanthramides are phenolic bioactive compounds (in more than 20 chemical forms), exclusive to oats, that exhibit anticancer properties against breast (MDA-MB-231) and human colon cancer cell lines, among others. In vitro anticancer studies of oats (grains) in malignant colon cell lines (HT-29) revealed that fermented (in the presence of *Lactobacillus acidophilus*) and non-fermented oats present high antioxidant and antiproliferative activity in colon cancer cells [99]. Avenanthramides have effects on the prevention and treatment of aging-related human diseases associated with oxidative stress and inflammation, including dermatological, cardiovascular, cerebrovascular, neurodegenerative, and metabolic diseases, and cancer [101]. Avenanthramides are produced by plants as a defense system against pathogens, have antioxidant properties 10 to 30 times greater than vanillin and caffeic acid, promote the activity of superoxide dismutase and glutathione peroxidase in rats fed them (20 mg Avn kg^−1^ body weight), also attenuate the production of free radicals induced by exercise (40 mg Avn kg^−1^ body weight), and have an antihypertensive effect (mediated by the production of nitric oxide) [105]. Avenanthramides are composed of N-cinnamoylanthranilic acids, anthranilic acid, and either cinnamic or avenalumic acid, and vary only in the pattern of substitution in the acidic moieties. Avenanthramides have been shown to significantly increase the expression of heme-oxygenase-1 (HO-1: phase II antioxidant enzyme) in KH-2 cells (adult human renal proximal tubule cells), in both a dose- (250–1000 μM) and time-dependent manner, by mediating reactive oxygen species (ROS) and stimulating the nuclear translocation regulated by Nrf2 (factor 2 related to nuclear factor E2); hydrogenation of the double bond of the carbonyl group in avenanthramides removes this effect. The antioxidant activity has been attributed to the hydroxyl groups of anthranilic and cinnamic acid of avenanthramides; without them, no DPPH radical scavenging has been detected, nor in the FRAP assay. In addition, the ortho-hydroxyl structure, present in cinnamic acid, is presumed to have a greater antioxidant capacity [106].

Form A, B, and C avenanthramides (Avn) are the main ones found in oats and oat bran, the concentration varying according to the processing applied; the Avn content in oat flakes has been reported to be 26–27 mg kg^−1^, while in bran it is around 13 mg kg^−1^ [102]. An enriched mixture of Avn (4, 20, and 40 ng mL^−1^; prepared from oat grains) preincubated (24 h) with human aortic endothelial cells significantly decreased the adhesion of monocytic U937 cells to IL-1β in a dose-dependent manner (evidence of antiatherogenic activity). Furthermore, the same mixture significantly suppressed the stimulated IL-1β expression of intracellular adhesion molecule-1 (ICAM-1), vascular cell adhesion molecule-1 (VCAM-1), and E-selectin, and secretion of the pro-inflammatory cytokine IL-6, chemokine IL-8, and monocyte chemoattractant protein (MCP)-1, inflammatory components involved in the formation of fatty portions in arteries. At the same time, the enriched mixture did not show toxicity to human aortic endothelial cells [107]. A possible anticancer mechanism (antiproliferative) of Avn (from oat grains: Avn, Avn-C, methylated derivative of Avn-C (CH3-Avn-C)) is by suppression of cyclooxygenase-2 (COX-2) and prostaglandin (PGE2) activity in colon endothelial cell macrophages [102]; they significantly inhibited the proliferation of human colon cancer cell lines (COX-2-positive HT-29, Caco-2, and LS174T, and COX-2-negative HCT116). The methylated extract of Avn-C had the most powerful anticancer activity (of those evaluated), presumably due to the presence of a single methyl ester group in the structure, which can increase lipid solubility and bioavailability, which means it is easily incorporated into the cell membrane and allows hindrance of the molecular pathways that participate in cell proliferation.

Some research regarding processed oat products has been published. Colloidal oatmeal has a long history of safe use in dermatology in the treatment of atopic dermatitis, psoriasis, drug-induced rash, and other conditions. Some in vitro and in vivo studies show some molecular mechanisms of the anti-inflammatory and antihistamine activity of colloidal oats [108,109]; however, the exact mechanism of action for the anti-inflammatory activity is unclear. The polar extracts of oats (methanol at 80%) significantly inhibited the expression of the cytokine IL-8 (an indicator of itching and prurism) [110]. The 80% acetone extract significantly inhibited levels of the inflammatory promoter NF-κB in the controls treated with TNF-α (25 and 50 μg mL^−1^) and the production of ROS (in keratinocytes). The groups of compounds recognized in the 80% methanol and 80% acetone extracts evaluated from oats include phenols and alcohol-soluble protein albumins. In the aqueous extract, there are water-soluble proteins such as globulins, and prolamines and carbohydrates, with important anti-inflammatory effects.

“Profermin” is a fermented vegetable product (water, fermented oats, barley malt, lecithin, and *Lactobacillus plantarum* 299v) used for the dietary treatment of ulcerative colitis; it is safe, well-tolerated, acceptable, and capable of reducing the simple clinical colitis activity index (SCCAI) to a statistically and clinically significant level in patients with mild to moderate flare-ups. The decrease in colitis (SCCAI) was 50% greater in the group consuming vegetable ferment (53%) than in the control group (Fresubin: 27%) [111]. A solution prepared with *Avena sativa* L. diluted in vinegar and hydroxyzine was evaluated in the reduction of uremic pruritus, a common complication in patients with chronic kidney disease. The solution significantly decreased the intensity of itching (2.03%), the consequences (4.82%), and the verbal descriptor (2.27%), but had no significant effects on the frequency (1.42%) or surface of the itch (12.40%) [112]. Four colloidal oat extracts were prepared with various solvents and tested in vitro for skin barrier-related gene expression and activity. Colloidal oats promoted the expression of target genes related to the skin barrier and resulted in the recovery of barrier damage in an in vitro model of atopic dermatitis. Clinically, the lotion improved skin dryness, hydration, and barrier [113]. The anti-irritation effect of oats on the skin could be mediated by polyphenols. Evidence indicates that the regular incorporation of oats into the diet can reduce the risk of some diseases associated with inflammation and microbial growth. In addition to these properties, the presence of fiber in oats contributes to reducing the risk of colon cancer.

## 3. Conclusions and Future Directions

Evidence indicates that representative crops from Hidalgo, Mexico, present important bioactivities which has been shown to reduce total and LDL cholesterol, improve HDL cholesterol, regulate blood pressure, enhance blood lipid profile, regulate glucose in postprandial blood, and insulin response, and reduce and maintain body weight. In addition, there is potential for the treatment of diseases associated with inflammation and microbial proliferation, as well as anticancer treatments. There are sources that help treat and/or prevent cardiovascular diseases and diabetes and improve immune functions by increasing immunoglobulins in the blood. Therefore, it is recommended to promote representative crops of the area, maintain their traditional use, and exploit them in innovative ways to produce compounds and foods beneficial to the health of the population. In addition, it is also worth highlighting that the crops reviewed are distributed throughout the world, where the benefits reviewed here can be used.

Once the bioactive benefits of crops are recognized, further development of plant improvements can be directed towards making plants more resilient to climate change, thereby supporting better control of the biosynthesis process of specific metabolites. Let us also remember that the biosynthesis routes of bioactive compounds (diverse depending on the type of compound) are a response to diverse stimuli specific to the plant, and that external stimuli from the environment also have an influence, thus requiring a constant recognition of the properties of the crops and their fruits, as the changes in the context also develop. The possible decline in plant production and its nutritional and bioactive quality will remain one of the worst consequences of climate change for years to come. With this, food security will be affected and will remain one of the main challenges for humanity. The challenge must be met with full knowledge of plant metabolism and its effects on the environment and humans. Moreover, that knowledge is still in continuous discovery, as demonstrated by the findings presented in this review. In addition to the inherent findings of plants, there are technological developments that can support plant food production, for the improvement of soils, crops, qualities, microbiological context, plant-microorganism relationships, and the effect of all of them on better qualities and higher quantities of phytochemicals. Some of the current tools to support the development of better crops are metabolomics and bioinformatics [2].

## Figures and Tables

**Table 1:** Main crops and their production in 2021, in the State of Hidalgo.

No.	Crop	Area (Ha)			Production (Tons)	Yield (Tons Ha^−1^)	Harvest
		Sown	Harvested	Loss	Obtained	Obtained	
1	Grain corn (white)	210,343.55	201,453.30	8890.25	618,156.85	3.07	Agricultural year
2	Green forage oats	24,161.78	24,049.78	112.00	324,378.96	13.49	
3	Grain barley	106,956.06	106,113.16	842.90	223,595.43	2.11	
4	Beans	17,420.18	17,381.18	39.00	13,338.79	0.77	
1	Green alfalfa	43,829.00	43,829.00	0.00	4,477,712.05	102.16	Perennial
2	Pulquero maguey (honey water: thousands of liters)	4842.20	1372.20	0.00	110,411.07	80.46	
3	Orange (Valencia)	5747.90	5478.50	0.00	65,627.47	11.98	
4	Cherry coffe	23,069.50	23,014.50	0.00	29,301.60	1.27	

Source: Own elaboration with data from the Agri-Food and Fisheries Information Service [5].

**Table 2:** Modification of the composition and/or bioactivity determined in alfalfa (*Medicago sativa* L.) because of different treatments.

Treatments	Conditions	Effects	References
Alfalfa sprout supplementation in a rabbit diet.	Ninety mixed white rabbits were fed for 50 days, divided into 3 groups: Standard (S) diet, Standard diet + 20 g d^−1^ of alfalfa sprouts (A), Standard diet + 20 g d^−1^ of flax sprouts (F).	The alfalfa sprouts presents increase in the total content of fatty acids (PUFA) (linoleic acid by 38.46% and linolenic acid by 70.05%), isoflavones (daidzein) in diets. The linolenic acid content in muscle of the alfalfa group was three times higher than control group. α-tocopherol content and α-tocotrienol, were up.	[12]
Dietary supplementation with alfalfa sprouts.	Dietary supplementation with alfalfa sprouts (40 g d^−1^) and quantification of bioactive compounds and cholesterol in chicken and chicken eggs.	Decreased cholesterol in chicken plasma from 79.2 to 65.2 mg dL^−1^ and in egg yolk from 11.5 to 10.4 mg g^−1^.	[13]
Sprouts alfalfa exposed to sound wave	Frequencies (250, 500, 800, 1000, and 1500 Hz) for two 1-h periods until 6 days.	Increase (24–50%) in the expression of genes that promote the production of L-ascorbic acid in sprouts (to 500 and 1000 Hz). The treatments increased the concentration of ascorbic acid and the antioxidant enzyme superoxide dismutase.	[14]
Substitution with alfalfa seed flour.	Adding alfalfa seed flour (0, 15, 30, 45% *w*/*w*) in rice flour biscuits (gluten free).	Increased linearly in: crude protein, total dietary fiber, total polyunsaturated, hardness, total phenolic content (22.9 to 112.9 mg GAE 100 g^−1^ DW for control and 45% substituted flour), and resistant starch. The antioxidant capacity increased proportionally from 14.7 to 194.6 μmol GAE 100 g^−1^ DW (FRAP) and from 739.3 to 3627.7 μmol TE 100 g^−1^ DW (ORAC), for control and 45% of alfalfa flour, respectively.	[11]
Different types of LED lighting in alfalfa sprout composition (FMC: fresh mass of cotyledons).	Four variants using cold white (10,032 K), warm white (3279 K), red green blue (RGB) LEDs with two chips activated: red and blue, which combined gave a violet colour.	Chlorophyll (a) up to 998.9 mg kg^−1^ in FMC with cold LED. β-carotene up to 44.6 mg kg^−1^ in FMC with red-green-blue LED (RGB). Chlorophyll a up to 843.3 mg kg^−1^ FMC, chlorophyll b up to 256.7 mg kg^−1^ FMC, β-carotene up to 21.6 mg kg^−1^ FMC, lutein up to 82.6 mg kg^−1^ FMC, neoxanthin up to 15 mg kg^−1^ FMC and violaxanthin up to 43.7 mg kg^−1^ FMC in shoots with sunlight. Total phenols up to 697 mg GAE kg^−1^ in FMC with blue LED (RGB). Ascorbic acid up to 155 mg kg^−1^ with sunlight.	[15]
Dry alfalfa sprouts (dry heat, freeze-dried).	Heat-dried samples (HD): stove at 60 °C for 24 h, and the freeze-dried samples (FD) for 24 h under vacuum (50 mTorr).	Greater decrease in isoflavone composition with the application of oven heat drying than with lyophilization. The lyophilisate increased the sterols concentration (41.82% for stigmasterol). The presence of carotenoids (zeaxanthin, β-carotene, retinol, lutein) was only detected after drying processes (not fresh).	[10]
Soak in water of seeds alfalfa.	Seeds were disinfected with hot water: soak at 85 °C, for 10 s; at 85 °C, for 40 s; at 90 °C, for 10 s; and at 100 °C for 10 s.	No effect on: lutein (23.4–26.6 mg kg^−1^), violaxanthin (16.0–17.2 mg kg^−1^), neoxanthin (3.5–4.1 mg kg^−1^), β-carotene (10.1–11.7 mg kg^−1^), total phenols (486.5–599.4 mg kg^−1^) and chlorophyll b (64.7–72.8 mg kg^−1^). The application of 100 °C caused a decrease in the content of ascorbic acid (from 84.5 a 67.5 mg kg^−1^) and a increased phenolic content (from 537.1 to 599.4 mg kg^−1^).	[16]
Supplementation, digestion and in vitro fermentation.	Adding alfalfa seed flour (0, 30, 45% *w*/*w*) in rice flour biscuits (gluten free). Simulated in vitro digestion and fermentation process.	Cookies with 30 and 45% of alfalfa seed flour presented the highest total phenolic content (0.42 and 0.56 mg g^−1^, respectively) (control 0.15 mg g^−1^). The in vitro fermentation of 8–48 h increased the concentration of lignans and phenolic acids, whose bioaccessibility at 24 h of in vitro fermentation were 16.2 and 12.2%, respectively.	[17]
Incorporation of selenium to alfalfa crop.	Inorganic selenium was used in two chemical forms: selenite (Na_2_SeO_3_) or selenate (Na_2_SeO_4_) (0, 20, or 200 μmol L^−1^).	Increase in anthocyanins in alfalfa (29%) after of 20 µmol L^−1^ selenite solution (≈8% reduction of DPPH).	[18]
Ultrasound in fresh alfalfa leaves.	Study factors and ranges: Solvent/raw material ratio (mL g^−1^): 5, 10, 15. Time (h): 1, 2, 3. Temperature (°C): 50, 65, 80. Power (W): 50, 100, 150. Ethanol concentration (%): 60, 75, 90.	Better yield (up to 1.61%) and bioaccessibility (up to 19.7%) of saponins. Conditions: solvent/raw material (9.5 mL g^−1^), extraction time (2.90 h), extraction temperature (79.1 °C), ultrasound power (111.0 W), ethanol concentration (88.2%).	[8]

**Table 3:** Modification of composition and/or bioactivity determined in *Agave salmiana* by different treatments.

Treatments	Conditions	Effects	Reference
Micropropagation	In vitro of plants from young germinated plantlets by axillary shoots.	Wild plants showed the highest phenolic content (13.06 mg EGA g^−1^). The antioxidant capacity was higher in vitro (369.84 µmol TE g^−1^ DW) than in normal ex vitro conditions (184.13 µmol TE g^−1^ DW) and with ex vitro irrigation (143.38 µmol TE g^−1^ DW) and than in wild conditions (130.39 μmol TE g^−1^ DW). Glycosylated flavanols were detected in plants with ex vitro irrigation (quercetin) and under normal ex vitro conditions (kaempferol). Saponins were detected: hecogenin (0.418–5.227 mg EHe g^−1^), tigogenin (18.821–31 mg EHe g^−1^), mannogenin (0.288–0.861 mg EHe g^−1^), and chlorogenin (0.339–2.042 mg EHe g^−1^).	[47]
	Micropropagation was from axillary shoots. Leaf tissue samples were taken from the in vitro plants, ex vitro acclimated plants obtained from open environment conditions, and plants obtained from a natural population.	The total phenolic acids were 35 and 40% higher in plants propagated in vitro (11.8 mg GAE g^−1^ DW) and ex vitro (10.8 mg GAE g^−1^ DW), compared with the wild type (7 mg GAE g^−1^ DW). The saponin content of plants in vitro (77.1 mg PE g^−1^ DW) and ex vitro (63.3 mg PE g^−1^ DW) were higher than those of wild type plants (2.1 mg PE g^−1^ DW). The antioxidant capacity (ORAC) of the plants in vitro (369 μmol TE g^−1^ DW) was higher compared to ex vitro and wild type (184 and 146 μmol TE g^−1^ DW, respectively).	[45]
	Hydrometanolic extraction was applied to the foliar tissues and the content of flavonols and saponins was analyzed.	The plants propagated in vitro presented a higher concentration of flavonols and saponins, quantifying 7 flavanols and 5 saponins. Herbacetin (most abundant flavonol found): wild plants (14.7 mg 100 g^−1^ DW), in vitro (16.3 mg 100 g^−1^ DW), in an open environment (38.4 mg 100 g^−1^). Tigogenin (most abundant saponin found and only detected in plants propagated): in vitro with 6895.2 mgPE 100 g^−1^ DW and 4997.8 mgPE 100 g^−1^ DW.	[48]
In vitro drought stress effect, generated by polyethylene glycol.	Stress medium: Murashige and Skoog (4.4 g L^−1^, pH 5.8, 30 g L^−1^ sucrose, and L2 vitamins) with polyethylene-glycol (0, 10, 20, 30%, 27 °C, photoperiod of 12:12 h light:dark, 60 days).	Plants grown with polyethylene glycol (30%) showed the lowest flavonol content, but the highest saponin content (tigogenin glycoside, 163 mg PE g^−1^ DW) and the highest antioxidant capacity (ORAC) (≈1000 mmol TE g^−1^ DW).	[49]
Ultrasonically-assisted supercritical fluid extraction (USFE).	Bagasse of *Agave salmiana* (part not indicated; 10 g). Process factors were pressure (150–450 bar), temperature (40–60 °C), and amount of co-solvent (5–10%).	Increased antioxidant capacity (FRAP) with the use of multiplate (US) transducer geometry of extracts at 20.91 μmol TE g^−1^ and saponin content at 61.59 μg g^−1^; comparing with the cylinder geometry (with 12.18 TE g^−1^ and 19.05 μg g^−1^, respectively).	[46]

**Table 4:** Composition and/or bioactivity determined in maize of different types (*Zea mays* L.) and applied treatments.

Corn	Treatments	Compounds and/or Products	Bioactivity	Reference
Mexican corn (13 pigmented grain): Arrocillo Amarillo (red, blue), Bolita (red, blue), Chihuahua Crystal Blue (blue, red), chalqueño corn (red, blue).	Nixtamalization with alkali (0.8% of the grain weight) for 30 min, followed by resting for 14–16 h, ambient drying, grinding, and sieving (0.5 mm).	Decreases the anthocyanin concentration of corn grains in the pericarp by 73 to 100%, varying according to the type of corn and portion.		[63]
Mexican corn (18 phenotypes).	White corn. Yellow corn.	Total phenolic content: - White corn: 170 mg GA 100 g^−1^ sample. - Yellow corn: 551 mg GA 100 g^−1^ sample. Total anthocyanin content: - White corn: 1.54 mg c3-G 100 g^−1^ sample. - Yellow corn: 70.2 mg c3-G 100 g^−1^ sample.	Antioxidant capacity: - Yellow corn: 89.4% by ABTS. - White corn: 26% by ABTS. - Red colored (phenotype Pinto): 100% by DPPH.	[65]
White, red, blue and purple corn (var Ver 42)	Ethanolic extracts (95%) from nixtamalized grains. Tortilla with nixtamalized grain.	The treatments reduced total phenols, and anthocyanins. Total phenolic content and anthocyanin (respectively): - Purple maize (1760 mg 100 g^−1^, 325.1 mg 100 g^−1^), - Red maize (465 mg 100 g^−1^, 82.3 mg 100 g^−1^), - Blue maize (343 mg 100 g^−1^, 63.1 mg 100 g^−1^), - White maize (170 mg 100 g^−1^, 1.59 mg 100 g^−1^).	The processing negatively affected the capacities of the grains. Quinone reductase induction (QR): purple > White > red > blue (anticancer activity). The purple genotype (Ver 42) and its products (dough and tortilla) showed the highest antioxidant capacity (70% by ABTS, 55% by PRAC) and QR (induction twice at 125 g mL^−1^).	[66]
Creole maize races (*Zea mays* L.) and pigmented varieties (yellow, red and blue).	Nixtamalization (alkaline boiling) and production of dough (grinding, drying) and tortillas.	Carotenoid content (μg of β-carotene eq g^−1^ extract) of raw maize grains and their products (masa, tortilla) respectively: - White (0.39, 0.17, 0.18) - Yellow (0.49, 0.28, 0.56) - Red (1.01, 1.14, 1.01) - Blue (0.18, 0.23, 0.22) For white grains, nixtamalization reduced carotenoids by 53 to 56%. Yellow grain suffered the highest losses from anthocyanins (174.44 to 10.30 mg of c3-GE 100 g^−1^ DW), not detectable in white maize and its products. The anthocyanin content of all grains was 174.44 to 963.00 mg of c3-GE 100 g^−1^ DW.	White corn (≈30%) and products (dough ≈20%, tortilla ≈25%) had higher antiradical (DPPH) activity than BHT (≈10% to 100 µM). Yellow corn (≈22%) and products (dough ≈18%, tortilla ≈29%) had higher antiradical activity (DPPH) than BHT (≈10% to 100 µM). Red (50%) and blue (40%) maize grain showed the highest antiradical activity. The antimutagenic activity (*S. typhimurium* TA98) of the grains: - White grain (35%) - Yellow (4 3%) - Red (53%) - Blue (56%)	[64]
18 samples of blue/purple grain of conical corn (EC), Chalqueño (CHAL), and Bolita (BOL) maize races.	40 grains without the germ, crushed, sieved (0.5 mm), and dried in an oven (40 °C, 18 h). Analysis extract by methanol (acidified to 1% with trifluoroacetic acid) and sonicated for 15 min.	Anthocyanins totals (AT) content (CHAL): varied from 579.4 to 1046.1 mg c3-GE kg^−1^ DW. The total soluble phenols (TSP) (CHAL): varied from 918.9 to 1479.2 mg GAE kg^−1^ DW. AT content (EC): varied from 997.8 to 1332.2 mg c3-GE kg^−1^ DW. TSP (EC): varied from 1328.6 to 1626.7 mg GAE kg^−1^ DW. AT content (BOL): varied from 304.1 to 528.0 mg c3-GE kg^−1^ DW. TSP (BOL): varied from 875.0 to 1276.2 mg GAE kg^−1^ DW.	Antioxidant activity (AA) (Chalqueño): 34 to 60.3% by DPPH. AA (Elote cónico): 46.6 to 60.4% by DPPH. AA (Bolita): 21.0 to 39.5% by DPPH.	[67]
Whole grains of 10 different colored corn (*Zea mays* L.) genotypes (landrace and an inbred line, over the year 2010).	Combined extracts: acetone/methanol/water (7:7:6, *v*/*v*/*v*), with alkaline hydrolysis and extracted with ethyl acetate and diethyl ether (1:1, *v*/*v*).	White and yellow corn: - Flavonoids (248.64 and 281.20 mg CE kg^−1^ DW), - β-carotene (0.21 and 0.70 mg kg^−1^ DW), - lutein (not detected and 5.91 mg kg^−1^ DW), - total phenolic content (5227.1 and 5393.2 mg GAE kg^−1^ DW), respectively. The light blue genotype had the highest content of total phenols (10,528.8 mg GAE kg^−1^ DW), flavonoids, and ferulic acid.	White and yellow showed antioxidant capacity (ABTS) between 15 and 20 mmol Trolox kg^−1^ DW. The light blue genotype had the highest scavenging activity (ABTS: 35.66 mmol Trolox kg^−1^ DW).	[68]
Blue corn flour	Nixtamalization. Maize grain in cooking (1:2, grain: water), 1.0% (*w*/*w*) of calcium hydroxide to 90 °C for 23 min, was soaked for 16 h at ambient temperature, was grounded, and passed through a flash dryer (260 °C for 4 s), the obtained flour was grounded in a mill using a hammer head and a 0.5 mm mesh screen.	Not change the resistant starch content or slow digestion.	The tortilla made with blue corn nixtamalized presented a lower glycemic index (58) and presented antioxidant capacity in the different fractions. They suggest a direct relationship between polyphenol content and antioxidant activity.	[69]
Spanish maize kernels, white (WF, Rebordanes variety), yellow (YF, Sarreaus variety) and purple (PF, Meiro variety).	Air-drying the maize kernels using a pilot-scale tray dryer (45 °C, 2 m s^−1^, 30% relative humidity, 5 kg m^−2^ of loading density, until an average maize moisture content of 11% DW), crushed, ground, and sieved (200 y 500 µm).	Total starch (TS, % *w*/*w*, DW) content of tested maize flours, yellow, white, and purple, ranged from 60.1 (whole flour 500 µm) up to 75.2 (purple 200 µm) and no clear differences between varieties were found.	No significant differences were observed among water desorption isotherms of maize varieties.	[70]
Five blue hybrid maize genotypes and Chalqueño and conic kernels were used as native genotypes cultivated in the highlands of Mexico.	Homogenized with 80% ethanol for 10 min, alkaline digestion (2 M NaOH), acidification (HCl), extraction with ethyl acetate.	The total anthocyanins and anthocyanins in free phenolics of the natives, chalqueño and conic are 646 and 892 mg c3-G kg^−1^ and 48.7 and 60.3%, respectively. The total anthocyanins and anthocyanins in free phenolics of the hybrid genotypes are in the range of 835–1052 mg c3-G kg^−1^ and 62.4–80.6%, respectively.	Antioxidant capacity (free and bound phenols, respectively): - Chalqueño (≈166 and ≈1600 mg TE kg^−1^). - Cónico (≈166 and ≈1700 mg TE kg^−1^). The range of antioxidant capacity of free and bound phenols of hybrid genotypes was 166–820 and 862–1533 mg TE kg^−1^, respectively.	[71]
White corn. Yellow corn.		White corn kernel (anthocyanin free). Yellow corn kernel (702 mg c3-GE kg^−1^)	Antioxidant capacity of: - White corn with 17.4 µM TE g^−1^ sample, - Yellow corn with 90% by ABTS.	[72]
Native Mexican blue corn (*Zea mays* L.).	Nixtamalization (maize kernels were cooked (1:3, maize grains/water) with 5.4 g of Ca(OH)_2_ L^−1^ water; 31 min, 85 °C, 8.1 h). Wet nixtamal was dried (55 °C/12 h), cooled, and milled to pass through an 80-US mesh (0.180 mm).	Increases the relative percentage of glycosylated anthocyanins and decreases acylated anthocyanins. The most abundant compounds (cyanidin-3-(6″-succinylglucoside) (Cy-Suc-Glu) and cyanidin-3-(6″-disuccinylglucoside) (Cy-diSuc-Glu)).		[73]
Blue and white cornmeal	Cooked samples were prepared in water (1:10 *w*/*v*) by a heating bath with shaking for 30 min.	Extractable polyphenols: - Blue maize flour (165 mg GA g^−1^ DW), - White maize flour (127 mg GA g^−1^ DW). Condensed tannins: - Blue maize flour (198.9 mg GA g^−1^ DW), - White maize flour (38.31 mg GA g^−1^ DW).	Antioxidant capacity and alpha-amylase inhibition (AAI): - Blue maize flour: 6.8 mg ET g^−1^ DW (DPPH), 13.1 mg ET g^−1^ DW (ABTS), 15.5 mg ET g^−1^ DW (FRAP), and 96.8% AAI. - White maize flour: 5.3 mg ET g^−1^ DW (DPPH), 11 mg ET g^−1^ DW (ABTS), 10.6 mg ET g^−1^ DW (FRAP), and 90.9% AAI. Increase in the total phenolic content and antioxidant capacity of cooked blue corn flours, compared to raw ones.	[60]
Purple corn grain flours (control: White corn)	Mixtures of various families (genotypes) of purple corn. Homogenized with ethanol (96%)/HCl (1 N) (85:15 *v*/*v*), 30 min.	White corn presented a total phenol concentration: 319 mg GAE 100 g^−1^. Total phenols (mixtures genotype) (range): 438 to 1933 mg GAE 100 g^−1^. Total phenols (original genotype): 1328 mg GAE 100 g^−1^.		[74]

**Table 5:** Composition and/or bioactivity determined in oat (*Avena sativa* L.).

Crop	Compounds and/or Products	Conditions of Bioactivity Detected	Bioactivity	Reference
Oat (no variety reported)	Oat bran concentrate containing 43% β-glucan.	Oat β-glucan hydrolysate was prepared by adding Celluclast (840 EGU g^−1^) to oat bran concentrate suspension (6.25% (*w*/*v*), 50 °C, pH 4.8).	Anti-cholesterol activity: reduced rat serum triglycerides, reduced weight gain, high-density cholesterol (HDL-C) in serum increased up to 42–62% and reduced low-density cholesterol (LDL) by 25–31%.	[89]
Oat (Derby variety)	β-glucan	Two β-glucan extracts were separately added to test solutions at concentrations of 0.1–0.5% (*w*/*w*). β-glucan fractions: 78.5% (E3, E4) content of extracts (*w*/*w*).	Decreased intestinal absorption of fatty acids (18:2 mainly). Inhibition of postprandial rise in glucose and insulin.	[90]
Oat (no variety reported)	β-glucan	Consumption in pigs of 3 and 6% in the diet.	Net glucose absorption reduction from 22 to 51%, relative to the intake percentage.	[91]
Oat (*Avena sativa* L.)	β-glucan	Dosage of 2000 mg kg^−1^ in reduction of hyperglycemia. Intake dose of 70 mg mL^−1^ for 6 weeks for enzyme inhibition.	Reduction of hyperglycemia. Inhibition of intestinal enzymes, sucrase (70.72%), maltase (83.33%) and lactase (89.43%), in diabetic mice. Similar protective effect to the diabetic mice as metformin (1% *w*/*v* metformin solution).	[92]
Oat (no variety reported)	β-glucan	Extract viscosity of 3 mPa, with the presence of starch of 40 g.	Glucose absorption reduction.	[93]
Oat (no variety reported)	β-glucan	Consumption of 3 g d^−1^ of oat or barley β-glucan is sufficient to decrease blood cholesterol.	There was a significant inverse relation in total cholesterol (−0.60 mmol L^−1^, −0.85 to −0.34), low−density lipoprotein (−0.66 mmol L^−1^, −0.96 to −0.36), and triglyceride/triacylglycerol (−0.04 mmol L^−1^, −0.15 to 0.07) after consumption of β-glucan.	[94]
Oat (*Avena sativa* L.)	Extract avenanthramides (EA) is: 6.07% N-(3′,4′-dihydroxycinamoyl)-5-hydroxyanthranilic acid, 4.37% N-(4′-hydroxycinnamoyl)-5-hydroxyanthranilic acid, 4.37% N-(4′-hydroxycinnamoyl)-5-hydroxyanthranilic acid, and 5.36% N-(4′-hydroxy-3′-methoxycinnamoyl)-5-hydroxyanthranilic acid. Phenols: vanillic acid (0.60%), caffeic acid (0.50%), syringic acid (0.54%), p-coumaric acid (0.16%), ferulic acid (0.08%), and sinapic acid (0.03%).	Mice in three experimental groups were (7th week) given EA at 250, 500, and 1000 mg (kg body weight)^−1^ d^−1^ by intragastric gavage (2 weeks). Mice were sacrificed and the liver was collected and stored until analysis.	Antioxidant effect against oxidative stress induced by D-galactose (50 mg kg^−1^ DW d^−1^) in mice, noted by increased antioxidant enzyme activity (dose-dependent mode) and the regulation of antioxidant gene expression.	[95]
Oat (no variety reported)	β-glucan (low molecular weight)	Concentration of 400 μg mL^−1^.	Deceased cancer cells viability (human pigmented malignant melanoma (Me45) and the human epidermoid carcinoma A431 cell line), while for the normal cells it was non-toxic.	[96]
Oat (no variety reported)	β-glucan	β-glucan (200 µg mL^−1^) with electroporation.	Antitumor activity due to decreased cell viability (human melanoma cell line (Me45)) of 12.5%. Not present toxic effects on normal cells.	[97]
Oat (no variety reported)	β-glucan (high and low molecular weight).		Decreased viability of cancer cells (human lung A549, H69AR) (about 50% decrease at 200 µg mL^−1^).	[98]
Oat (*Avena sativa* L.)	Avenantramide	100 μL of *Lactobacillus acidophilus* was added to finely powdered oats (solution 1 g/50 mL water) for fermented oats. And control was measured (non-fermented).	In vitro studies revealed that fermented and non-fermented oats displayed higher antioxidant activity, having a corresponding IC_50_ value of 201.03 μL and 236.46 μL, respectively. The colon cancer cell (HT29) death percentage, varied in the range of 41.81% and 87.48%, with the highest cytotoxic activity being for non-fermented oats (25 µg mL^−1^).	[99]
